# Prevalence of *Enterococcus* spp. and the Whole-Genome Characteristics of *Enterococcus faecium* and *Enterococcus faecalis* Strains Isolated from Free-Living Birds in Poland

**DOI:** 10.3390/pathogens12060836

**Published:** 2023-06-16

**Authors:** Renata Kwit, Magdalena Zając, Aleksandra Śmiałowska-Węglińska, Magdalena Skarżyńska, Arkadiusz Bomba, Anna Lalak, Ewelina Skrzypiec, Dominika Wojdat, Weronika Koza, Emilia Mikos-Wojewoda, Paulina Pasim, Milena Skóra, Marcin Polak, Jarosław Wiącek, Dariusz Wasyl

**Affiliations:** 1 Department of Microbiology, National Veterinary Research Institute (PIWet), 24-100 Pulawy, Poland; aleksandra.smialowska@piwet.pulawy.pl (A.Ś.-W.); magdalena.skarzynska@piwet.pulawy.pl (M.S.); anna.lalak@piwet.pulawy.pl (A.L.); ewelina.skrzypiec@piwet.pulawy.pl (E.S.); dominika.wojdat@piwet.pulawy.pl (D.W.); weronika.koza@piwet.pulawy.pl (W.K.); emilia.mikos@piwet.pulawy.pl (E.M.-W.); paulina.greszata@piwet.pulawy.pl (P.P.); milena.skora@biol.uni.lodz.pl (M.S.); wasyl@piwet.pulawy.pl (D.W.); 2 Department of Omic Analyses, National Veterinary Research Institute (PIWet), 24-100 Pulawy, Poland; arkadiusz.bomba@piwet.pulawy.pl; 3 Department of Zoology and Nature Protection, Institute of Biological Sciences, Maria Curie-Sklodowska University (UMCS), 20-033 Lublin, Poland; marcin.polak@mail.umcs.pl (M.P.); jaroslaw.wiacek@mail.umcs.pl (J.W.)

**Keywords:** wild birds, *Enterococcus faecium*, *Enterococcus faecalis*, antimicrobial resistance, WGS

## Abstract

Enterococci as opportunistic bacteria are important for human health. Due to the prevalence and ease of acquisition and transfer of their genes, they are an excellent indicator of environmental contamination and the spread of antimicrobial resistance. The aim of the study was to assess the prevalence of *Enterococcus* spp. in wild birds in Poland, determination of antimicrobial susceptibility and WGS analysis of *Enterococcus* (*E*.) *faecium* and *E. faecalis*. For this purpose, 138 samples from various species of free-living birds were tested, with 66.7% positive results. Fourteen species were detected, with *E. faecalis* being the most common, followed by *E. casseliflavus* and *E. hirae*. In antimicrobial susceptibility testing, 10.0% of *E. faecalis* and 50.0% of *E. faecium* showed resistance to one antimicrobial agent, in addition the MDR phenotype which was found in one *E. faecium*. The most common resistance phenotype included tetracycline and quinupristin/dalfopristin. The WGS analysis confirmed the significant advantage of the virulence gene diversity of *E. faecalis* strains over *E. faecium*. In addition, plasmid replicons were found in 42.0% of *E. faecalis* and 80.0% of *E. faecium*. The obtained results confirm free-living birds can be a reservoir of *Enterococcus* spp. with a considerable zoonotic potential.

## 1. Introduction

Enterococci, the bacteria first described in 1899 by Thiercelin, were originally included in the genus *Streptococcus*, and only in 1984 were distinguished as a separate genus *Enterococcus* [1]. Currently, over 60 species belonging to the genus *Enterococcus* are known, with high variability in terms of biochemical and morphological characteristics, as well as preferences for occupied niches [2,3]. Thanks to their high tolerance of different environmental conditions, they are commonly isolated from plants, soil, water, sewage and food [1,4,5,6,7,8].

As a component of the natural intestinal flora, they usually do not cause disease and their presence benefits the host, thanks to their probiotic effect [4,9]. However, as opportunistic microorganisms, in the event of a decrease in host immunity, they can lead to pathological conditions such as: sepsis, bacteremia, endocarditis, wound infections, urinary tract infections and others [2,4,9,10].

These bacteria, having numerous determinants of invasiveness and pathogenicity, pose a great challenge in the hospital environment, especially the species *Enterococcus* (*E.*) *faecium* and *E. faecalis* [1,2,4,9]. In addition, enterococci are naturally resistant to many antibiotics. In addition, these microorganisms are characterized by a high ability to exchange genes responsible for pathogenicity and resistance to antibacterial substances [1,4,9,10,11,12]. Transfer of mobile genetic elements can take place both within and outside the genus [4,13,14,15].

Wild birds seem to be an important reservoir of enterococci. Inhabiting areas overlapping with human agricultural or recreational areas, they may be in constant contact with human and farm animal pathogens, including highly dangerous bacteria resistant to many antimicrobial agents [1,5,13]. Available studies suggest a two-way, indirect or direct, transfer of microorganisms between wild and farmed animals as well as humans [2,4,10,11,13,16]. Due to their lifestyle and the migrations associated with it, birds can become a vector of the microbe over very long distances [1,10,13,16,17]. Contact between individuals using the same habitats, common migrations and preferred type of nutrition play an important role in infection and the transfer of pathogens between seemingly unrelated niches [1,5]. Due to the lack of antibiotic therapy in free-living birds, the condition of their intestinal microbiota may reflect the level of environmental contamination with drug-resistant bacterial strains [1,10,16].

The aim of this study was to assess the occurrence of enterococci and establish a genomic characterization of *E. faecium* and *E. faecalis* isolated from fecal samples, crop swabs and pellets from a range of bird species occurring in south-eastern Poland. This study provides important information filling the knowledge gap on the role of wild birds in the spread of pathogens and antimicrobial resistance determinants.

## 2. Materials and Methods

### 2.1. Sample Collection

**Figure 1:**
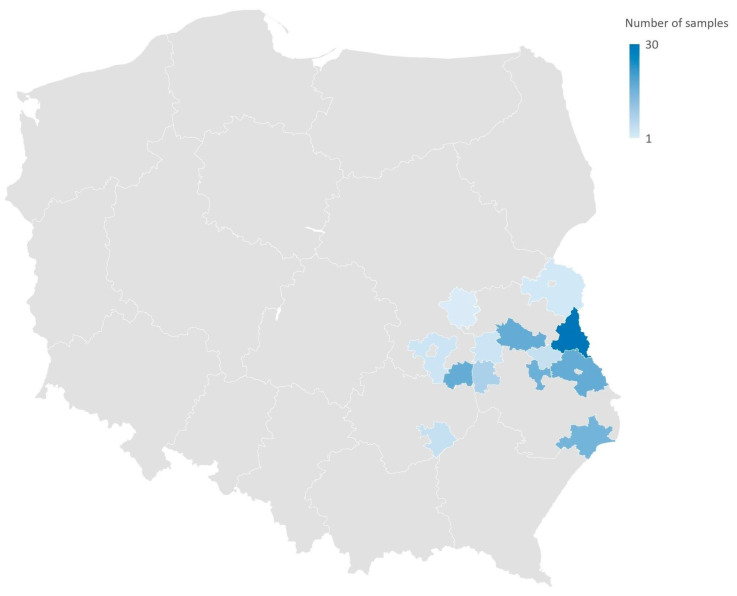
Geographical distribution (commune level) of wild birds sampling. The samples were collected from nature from nests surrounding or during the ringing activity of wild individuals.

A total of 138 samples (105 feces, 27 pellets, 6 crop swabs) from 20 species of free-living birds were collected between 2020 and 2021: White stork (*Ciconia ciconia*, *n* = 58), Barn swallow (*Hirundo rustica*, *n* = 15), Common crane (*Grus grus*, *n* = 11), Greylag goose (*Anser anser*, *n* = 7), Mute swan (*Cygnus olor*, *n* = 7), Common blackbird (*Turdus merula*, *n* = 5), Mallard (*Anas platyrhynchos*, *n* = 5), Common house martin (*Delichon urbicum*, *n* = 4), Song thrush (*Turdus philomelos*, *n* = 4), Great tit (*Parus major*, *n* = 3), Spotted flycatcher (*Muscicapa striata*, *n* = 3), Black redstart (*Phoenicurus ochruros*, *n* = 2), Common chaffinch (*Fringilla coelebs*, *n* = 2), Common woodpigeon (*Columba palumbus*, *n* = 2), Eurasian blackcap (*Sylvia atricapilla*, *n* = 2), Icterine warbler (*Hippolais icterina*, *n* = 2), Lesser whitethroat (*Curruca curruca*, *n* = 2), European greenfinch (*Chloris chloris*, *n* = 1), European robin (*Erithacus rubecula*, *n* = 1), and Fieldfare (*Turdus pilaris*, *n* = 1). The convenience sampling took place in the birds’ natural environments in south-eastern Poland (Figure 1) and was carried out by ornithologists from the Department of Zoology and Nature Protection, UMCS, during the ringing activity or nest inspection. The study fulfilled the current Polish law and was permitted by the Ministry of the Environment (approval number: DL-III.6713.11.2018.ABR) and the General Directorate for Environmental Protection (approval number: DZP-WG.6401.102.2020.TŁ). The Regional Directorate for Environmental Protection (RDOŚ) in Lublin allowed for the research project through a letter (approval number: WPN. 6401.6.2018.MPR). The samples were referred to the National Reference Laboratory for Antimicrobial Resistance (NRL) at PIWet for laboratory analyses.

### 2.2. Isolation and Identification

Samples were pre-enriched in buffered peptone water (BioMaxima S.A., Lublin, Poland, 1:10 *v*/*w*; 18 ± 2 h at 37 ± 1 °C) followed by selective isolation and differentiation on BEAA medium (Bile Esculin Azide Agar ISO 15788:2009) or CHROMagar Orientation (BioMaxima S.A., Lublin, Poland) differentiating a wide spectrum of microorganisms. After overnight incubation at 37 ± 1 °C, 3 colonies from each plate with a characteristic morphology (BEAA: small gray colonies on the darked of the medium around the growth, CHROMagar Orientation: turquoise colonies) were selected and transferred to nutrient agar (Oxoid, Hampshire, United Kingdom) which was incubated overnight at 37 ± 1 °C.

The identification of microorganisms was performed using the MALDI TOF MS technique (Matrix Assisted Laser Desorption and Ionisation Time of Flight Mass Spectrometry) and the MALDI Biotyper (MBP COMPASS 4.1). All samples were prepared in accordance with the Standard operating procedure formic acid extraction (EX) method (Brucker Daltonic GmbH, Revision 3, August 2013). Obtaining a log score (log(score)) in the range of 2000–3000 was considered to give a high degree of confidence of the best match to the reference species in the database. Strains belonging to the genus *Enterococcus* were preserved using LB medium with 15% glycerol (POCh, Gliwice, Poland) and stored at −20 °C until further analysis.

### 2.3. Antimicrobial Susceptibility Tests

A microbroth dilution method (Sensititre EU Surveillance Enterococcus EUVENC AST plate, Thermo Fisher Scientific Waltham, MA, USA) was used for determine the MIC (minimal inhibitory concentration) values for the following antimicrobials: vancomycin (VAN 1–128 mg/L), teicoplanin (TEI 0.5–64 mg/L), quinupristin/dalfopristin (SYN 0.5–64 mg/L), tetracycline (TET 1–128 mg/L), daptomycin (DAP 0.25–32 mg/L), ciprofloxacin (CIP 0.12–16 mg/L), erythromycin (ERY 1–128 mg/L), tigecycline (TGC 0.03–4 mg/L), linezolid (LZD 0.5–64 mg/L), gentamicin (GEN 8–1024 mg/L), ampicillin (AMP 0.5–64 mg/L) and chloramphenicol (CHL 4–128 mg/L).

Antimicrobial susceptibility testing was carried out for *E. faecium* (n = 10) and *E. faecalis* (n = 50). Briefly, from a fresh culture on nutrient agar (Oxoid), a suspension was made with a density of 0.5 on the McFarland scale in 0.9% NaCl (bioMerieux, Marcy-l’Étoile, France) from which 30 µL suspension was transferred to 11 mL of Mueller Hinton broth (ThermoFisher Scientific, Waltham, MA, USA), and after thorough vortexing, 50 µL of suspension was added into each well of the plate followed by overnight incubation (37 ± 1 °C). *Enterococcus faecalis* ATCC 29212 and *Staphylococcus aureus* ATCC 29213 (https://www.atcc.org, accessed on 11 November 2022) were used as quality control strains. The MICs were read with Sensititre™ Vizion™ reader (Thermo Fisher Scientific Waltham, MA, USA) and interpreted using epidemiological cut-off (ECOFF) values provided by The European Committee on Antimicrobial Susceptibility Testing—EUCAST (https://www.eucast.org (accessed on 18 April 2023)). A strain was regarded as resistant (non-wild type, NWT) when an MIC value above the cutoff was noted. Strains with MIC values below the ECOFF were recognized as susceptible (wild type, WT). Strains resistant to at least 3 antimicrobial classes were identified as multidrug resistant (MDR).

### 2.4. Whole-Genome Sequencing

The strains belonging to *E. faecalis* (*n* = 50) and *E. faecium* (*n* = 10) were subjected to whole-genome sequencing. DNA extraction was prepared from nutrient agar plate cultures with a Maxwell^®^ RSC Cultured Cells DNA Kit—Automated DNA Purification from Mammalian and Bacterial Cultured Cells (AS1620 Promega, Madison, WI, USA), according to the manufacturer’s instructions, with a Maxwell^®^ RSC Instrument (Promega, Madison, WI, USA). Sequencing libraries were prepared with KAPA HyperPlus (Roche, Pleasanton, CA, USA) according to the manufacturer’s protocol. Whole-genome sequencing was performed on the NextSeq sequencer (v2.5 2 × 150 bp, Illumina, San Diego, CA, USA).

### 2.5. Bioinformatic Data Analysis

FastQC 0.11.5 was used for the raw reads quality check. Nextseq raw reads were trimmed using fastp 0.23.0 (https://github.com/OpenGene/fastp accessed on 8 August 2022) and assembled by SPAdes 3.15.3 [18]. AMR genes were found using AMRFinderPlus 3.11.2 (https://github.com/ncbi/amr accessed on 24 May 2022) with specified ‘--plus’ and --organism’ options and ResFinder (https://cge.food.dtu.dk/services/ResFinder accessed on 24 May 2022) Update: software: 8 August 2022, database: 24 May 2022). AMRFinderPlus v3.10.24 database version was 2022-12-19.1. Plasmid replicons were identified using ABRicate 1.0.1 with PlasmidFinder database accessed on 11 January 2023. MLST was conducted using mlst 2.19.0 (https://github.com/tseemann/mlst, accessed on 24 May 2022). Sequences of enterococci with unknown STs were submitted to pubMLST to assign new sequence types using the MLST algorithm [19,20]. CSI Phylogeny 1.4 (call SNPs and infer phylogeny) CGE with input parameters—minimum depth at single nucleotide polymorphism (SNP) positions: 10, relative depth at SNP positions: 10, minimum distance between SNPs (prune): 10, minimum SNP quality: 30, minimum read mapping quality: 25, minimum Z-score: 1.96—was applied for phylogeny tree preparation [21]. Construction of the trees was carried out using the reference genomes of, respectively, *Enterococcus faecium* ATCC19434 and *Enterococcus faecalis* ATCC29212 (https://www.atcc.org, accessed on 11 January 2023). The online tool iTOL v5 was applied for phylogeny tree visualization [22].

## 3. Results

### 3.1. Enterococcus Prevalence

Overall, 66.7% (*n* = 92) of the samples were found positive for *Enterococcus* spp., whereby of which 128 *Enterococcus* isolates were isolated and differentiated into 14 different species. 

**Figure 2:**
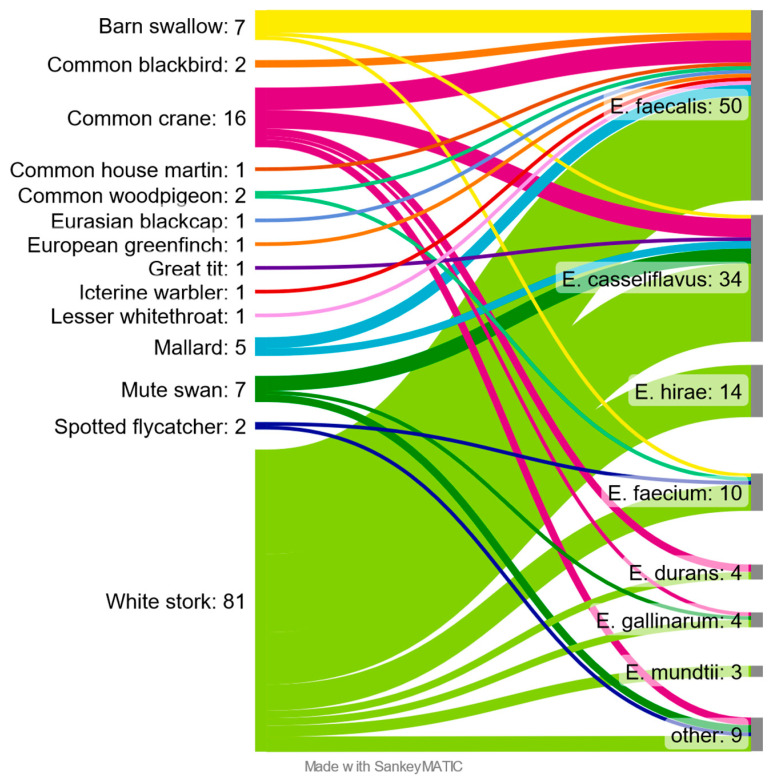
Enterococcus species found in different species of free-living birds.

*E. faecalis* was the most common and obtained from 50 samples, which constituted 39.1% of positives. The second most common was *E. casseliflavus* with 26.6% (*n* = 34) followed by *E. hirae* 10.9% (*n* = 14). *E. faecium* was detected in 10 (7.8%) positive samples. The remaining species together accounted for 15.6% (*n* = 20). Correlations between the sampled species of birds and isolated species of bacteria of the genus *Enterococcus* were presented in the Sankey diagram (Figure 2).

Among the birds studied, the largest number and diversity of *Enterococcus* species were found in the White stork and Common crane, with 81 and 16 confirmed isolates, respectively. Among the White stork samples, only seven were found negative, while all Common crane samples were positive. *Enterococcus* bacteria were not isolated from samples taken from Black redstart, Common chaffinch, European robin, Fieldfare, Greylag goose and Song thrush. “Other” shown on the diagram contain two isolates of *E. aquimarinus* from Mute swan, two isolates of *E. radioresistens* and single isolates of *E. cecorum* and *E. canintestini,* from White stork, *E. hermanniensis* and *E. thailandicus* from Common crane and *E. haemoperoxidus* from Spotted flycatcher.

### 3.2. Antimicrobial Resistance Results

The distribution of the Minimal Inhibitory Concentrations (MICs) of the tested *E. faecium* and *E. faecalis* are summarized in Table 1 and Table 2, respectively.

Six *E. faecium* showed resistance to quinupristin/dalfopristin, of which one isolate from White stork that was additionally resistant to other tested active substances obtained high MIC values for chloramphenicol, ciprofloxacin, erythromycin, gentamicin and tetracycline, and thus was considered multidrug-resistant (MDR). No resistance was observed for ampicillin, linezolid, daptomycin, tigecycline, teicoplanin and vancomycin. Five *E. faecalis* isolates were characterized by high MIC values for tetracycline (White stork *n* = 4, Mallard *n* = 1). For remaining substances such as ampicillin, chloramphenicol, ciprofloxacin, erythromycin, gentamicin, daptomycin, tigecycline, teicoplanin, quinupristin/dalfopristin and vancomycin, no resistance was found. No MDR *E. faecalis* isolates were noted.

### 3.3. Genetic Characteristics of E. faecium

Multilocus sequence typing (MLST) revealed 10 STs among all 10 sequences with three being the new ones (ST2340, ST2341, ST2342). The novel STs were listed in Supplementary Table S1. ST2341 was characterized by a new *purK* allele (*purK*(167)), while the remaining two resulted from new combinations of the known alleles. The minimum SNP dissimilarities were observed between strains belonging to ST22 (754B) and ST32 (682B_2) (1565 SNPs) and the highest difference of 28 502 SNPs was noted between strains belonging to ST54 (678B_3) and ST2342 (P_20_BEAA_132B).

**Figure 3:**
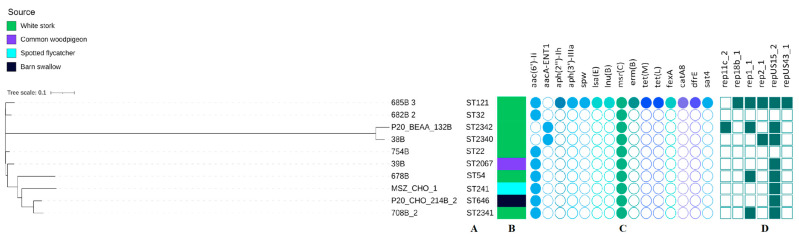
Phylogenetic tree of *E. faecium* isolates found in different species of migratory birds (sequence types—A, source of isolation—B, map of resistance genes—C, plasmid replicons—D). Full and empty circle or square mean presence and absence of antimicrobial resistance (AMR) gene or plasmid replicons, respectively.

The whole-genome sequencing data revealed the occurrence of at least two antimicrobial resistance genes in all *E. faecium* (Figure 3). The *msr*(C) gene was present in all strains, while the *aac(6′)-Ii* gene was carried by eight isolates and only two contained the *aacA-ENT1* gene. The White stork isolate (685B_3) was the only one carrying the multiple genes coding resistance to several antimicrobial classes such as quinolones, aminoglycosides, macrolides, streptogramins, lincosamides, tetracyclines, phenicols, and folate pathway antagonists. Resistance to ciprofloxacin was confirmed in strain 685B 3 and, additionally, the ResFinder analysis was performed for point mutations in the QRDR (quinolone resistance-determining region) of the *parC* and *gyrA* genes. Amino acid changes were detected in codon 80 of the *parC* allele (serine changed to arginine), and codon 87 of the *gyrA* allele (glutamic acid changed to glycine), confirming the phenotype. In addition, the presence of the *aac(6′)-Ie-aph(2″)-Ia* gene (100% identity) was also detected, which was not shown using AMRFinderPlus 3.11.2.

Eight of the ten *E. faecium* strains contained at least one plasmid replicon (namely the most common repUS15_2), while five of them contained two up to five (685B_3) different plasmid replicons.

Only two genes encoding virulence factors were detected: *acm* and *efaAfm*. Each strain contained the *efaAfm* gene, while the *acm* gene was found in seven of the isolates (Figure 3).

### 3.4. Genetic Characteristic of E. faecalis

Among *E. faecalis* isolates, 39 different types of ST have been found, of which 11 were described for the first time: ST1366, ST1368-1371, ST1373-1378 (Supplementary Table S2). Among the new STs, seven were distinguished by the presence of a new alleles ST1368 (*pstS*(121)), ST1369 (*yqiL*(118)), ST1370 (*xpt*(111)), ST1371 (*aroE*(126)), ST1375 (*pstS*(122)), ST1376 *pstS*(123)), ST1377 (*xpt*(112)). Remaining STs showed just new allele patterns that have not yet been reported. The most common ST was ST21 (*n* = 3). ST40, ST81, ST232, ST274, ST482, ST579, ST648, ST749, ST1366, ST1370 were each detected twice, while the remaining STs occurred in single isolates.

The phylogenetic variation between tested strains peaked up to 19,576 SNPs. Two ST 1370 strains isolated from Common crane and White stork were indistinguishable. Differences of 3 SNPs were noted between ST1366 isolates while ST579 strains differed by 71 SNPs. Variations between the remaining isolates were above 100 SNPs.

**Figure 4:**
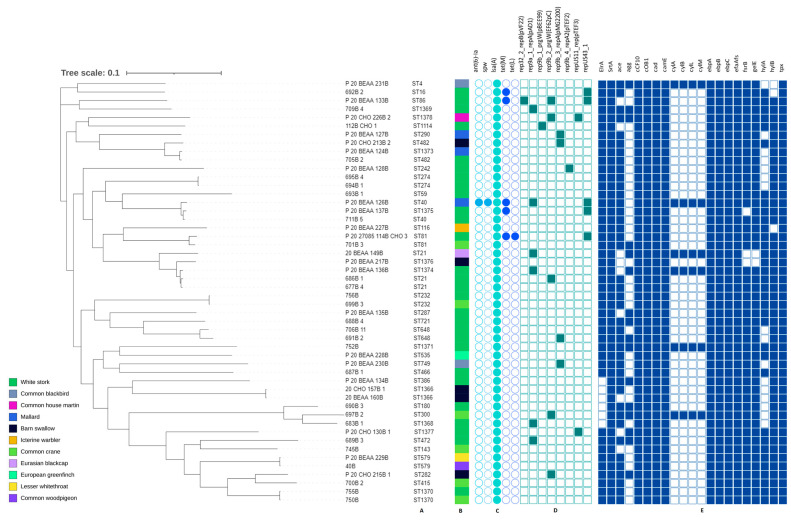
Phylogenetic tree of *E. faecalis* isolates found in different species of migratory birds (sequence types—A, source of isolation—B, and map of resistance genes—C, plasmid replicons—D and virulence determinants—E). Full and empty circle or square mean presence and absence of gene or plasmid replicons, respectively.

The results of *E. faecalis* sequence analysis are shown in Figure 4. The presence of the *lsa(A)* gene was confirmed in each *E. faecalis*. The *tet*(M) gene was detected in all tetracycline-resistant isolates (10.0%); one of them additionally carried the *tet*(L) gene. One strain harbored the *ant(6)-Ia*, *spw* genes but their phenotypic expression was not tested.

Twenty-one (42.0%) isolates carried eight different plasmids replicons, with the number per strain varying between one (mostly), and three (single P_20_BEAA_133B strain). The most common plasmid replicon was rep9a_1_repA(pAD1) (12.0%) followed by rep9b_2_prgW(EF62pC) and repUS43_1 (10.0% each).

All *E. faecalis* strains tested contained genes encoding virulence factors. The most common were: *SrtA, cCF10, coOB1, cad, camE, ebpA, ebpB, ebpC, efaAfs* and *tpx*, present in each isolate. The presence of *hylB, gelE* (96.0%) and *fsrB* (94.0%) was reported less frequently, while *cylA, cylB, cylL* and *cylM* occurred occasionally (12.0%). One of the strains (752B) contained all the above mentioned genes. The presence of virulence genes in the remaining microorganisms was in the range of 61.9–95.2%.

## 4. Discussion

Enterococci, initially considered to be commensals, have gained wide interest as opportunistic organisms capable of causing numerous pathological conditions, precisely in hospital environments. In our work, we present the prevalence and diversity of the genus *Enterococcus* among wild birds of eastern Poland, antimicrobial resistance and its background, as well as some genomic features of *E. faecalis* and *E. faecium*.

As in other reports, *E. faecalis* was the dominant species in our study [1,5,16,17,18]. *E. casseliflavus* and *E. hirae* were determined less frequently. *E. faecium*, which in many studies is listed as the second after *E. faecalis* [1,16], or as the most frequently isolated species from wild birds [10,13,18,19,20], was only the fourth often observed in our dataset. Differences in the *Enterococcus* spp. occurrence may be due to significant discrepancies in the detection methods, species of birds studied, geographical representativeness and season during which samples were taken. Interestingly, Stępień-Pysniak et al. did not confirm any *E. faecalis* in 52 samples from free-living birds, despite studies conducted on avifauna living in an area similar to our study [19]. *E. casseliflavus, E. hirae* and *E. durans* were previously found in wild birds both in Poland [19] and other countries [1,10,13,16,18]. The greatest diversity and number of strains was observed in White stork, Common crane and Barn swallow, which results directly from the largest number of samples taken.

Great variability in the observed STs, including newly described variants, indicates a huge diversity of bacterial populations. However, *E. faecium* ST22 described in this study has previously been reported to occur in livestock [21] and, similarly to ST54, in human clinical and screening isolates [22]. *E. faecium* ST241 has previously been found among a variety of *Enterococcus* isolates from the Coomera River in Australia [23]. Strains assigned to ST32 are common and have been associated with isolates of various origins, including meat samples from chickens [24], non-hospitalized persons, hospitalized patients, husbandry and the environment [25].

*E. faecalis* strains belonging to ST21 were confirmed in two isolates from the White stork and one from the Eurasian blackcap. The same ST has also been determined in the Eurasian marsh harrier (*Circus aeruginosus*) in another study [26]. In addition, isolates belonging to ST21 have been associated with *Enterococcus* isolated from both wild and farmed animals [2,21], as well as hospitalized patients, non-hospitalized patients, the environment and animal food [25]. Here, we described five *E. faecalis* STs (ST16, ST232, ST287, ST290, ST749) which have already been occurring among avifauna in Poland [17], while some others (ST4, ST16, ST40, ST81) have occurred in Spain [27]. Moreover, ST16 has also been detected in Cattle egret (*Bubulcus ibis*) from Tunisia [1] and in crows in the USA [20]. Of note, many of the sequence types of enterococci identified in this study were also reported in Polish hospitals [28]. Other identified sequence types in this study, according to data available in the pubMLST, have also been isolated from hospitalized and non-hospitalized patients, environment, husbandry, animals [25] and yolk sac infections in broiler chicks [29].

The high diversity of ST occurring in the studied species of birds and the common occurrence in diverse environments confirm the fact that bacteria of the genus *Enterococcus* are well-adapted to a broad range of hosts.

This work embraced bacteria derived from wild animals which have probably not been exposed to antimicrobial treatment. However, as synanthropic species adapted to life in areas inhabited by humans, the animals are likely exposed to contact with drugs and their residues in urban or agriculture areas. Despite this, a low occurrence of resistant microorganisms was noted, since as few as just 11 out of 60 strains (18.3%) showed resistance to any of the tested antimicrobials, and resistance patterns covered mostly no more than one substance. This is quite diverse from other studies reporting a much higher frequency of resistant bacteria ranging 46.4–100% [1,13,16,17,19,27]. The diet, behavior and habitat of birds greatly influence the incidence of resistant enterococci. Synanthropic birds and raptors are especially vulnerable due to the wide range of food, feeding habits and proximity contact to humans [5,10,13,20].

A review of the current literature confirms the widespread occurrence of MDR enterococci in avifauna in various parts of the world [1,10,16,17,19,20]. In our study, one *E. faecium* isolated from a White stork (685B) was resistant to more than three antimicrobials. In addition, it also carried *aph(3′)-IIIa, dfrE, sat4*, and *lnu(B)* genes encoding resistance to kanamycin, trimethoprim, streptothricin and lincosamide, respectively. Unfortunately, these substances were not tested in the current study.

The phenotypic resistance profile of the MDR strain can be compared to the resistance patterns described in farm animals, most often showing resistance to aminoglycosides, tetracyclines and macrolides [30,31,32,33]. This is due to the high consumption of these substances in animal production, which is particularly visible in the case of tetracycline [10,30,34]. In our study, resistance to tetracycline was one of the most common, and similarly to other studies, it resulted from the presence of the *tet(M)* and *tet(L)* genes [1,10,16,17,19,20]. Resistance to erythromycin among wild bird isolates is common in many countries and ranges from 34.2% to 81.0% [1,5,13,17,18,19], and the *erm(B)* gene is the most frequently described determinant of resistance to this agent [1,16,18,19,35]. Similarly to *aac(6’)-Ii*, the *msr(C)* gene found in all *E. faecium* is common and species-specific [32,36]. All *E. faecalis* carried the *lsa(A)* gene encoding intrinsic resistance to clindamycin and quinupristine-dalfopristin [32]. The phenotypic resistance of *E. faecium* to quinupristine-dalfopristine has not been confirmed by the presence of the most commonly associated genes *vat(D)* and *vat(E)*, which may suggest the presence of other resistance mechanisms [37]. *Enterococcus* spp. are naturally resistant to low concentrations of aminoglycosides. However, in many cases, strains show resistance classified as HLAR (high-level aminoglycoside resistance) [10], obtaining high MIC values. In our isolate, resistance to gentamicin was associated with the presence of the often-described gene *aac(6’)-Ie-aph(2″)-Ia* [1,13,18,35,38]. In addition, a number of other determinants encoding enzymes capable of modifying this class of drugs was also found: *aph(3’)-IIIa, aph(2″)-Ih, aac(6’)-Ii, spw, sat4* [13,18,19,35]. In addition, in one of the *E. faecalis* isolates, the presence of the *ant(6’)-Ia* gene associated with a high degree of resistance to streptomycin was confirmed [10,13,19,35]. The HLAR phenomenon is of concern because it reduces the chances of successful treatment of infections enterococci by means of a combination of aminoglycosides with active substances acting on the cell wall [10]. Our results indicate no resistance to vancomycin, ampicillin, linezolid, teicoplanin, daptomycin and tigecycline antimicrobial agents used to treat invasive infections caused by *Enterococcus* spp. [32]. Although, such cases have been previously described in free-living birds in Poland and in the world [1,10,13,16,17,20,35]. Taking into account that enterococci can carry resistance genes and transfer to other pathogens, often more than even a single MDR of a strain in wild bird samples may be of concern.

There are few papers providing knowledge about enterococcal virulence factors in enterococci originating from free-living birds, and those that address this topic do not present a complete picture of the issue [1,18,27,35]. This study confirmed a high percentage of isolates containing multiple virulence genes, which is characteristic of clinical strains. It also shows a significant advantage of virulence gene diversity in *E. faecalis* strains over *E. faecium*, which is also described by other authors [8,31,37,39]. Most of the virulence factors encoded by the genes found in this study (*agg, efaAfs, efaAfm, ace, acm, ebpA, ebpB, ebpC*) are involved in adhesion to host tissues, which is the first stage of biofilm formation, urinary tract infections and endocarditis [8,9,37,40,41,42]. One of the most frequently mentioned surface virulence determinants of great importance in the development of infection is the aggregating substance (*agg* gene) which additionally protects cells against lysosomes by increasing their hydrophobicity and enables conjugative plasmid transfer, which also promotes the spread of antibiotic resistance [9,43]. Another important gene, found in 82.0% of *E. faecalis*, is the *ace* gene, which determines the formation of the type I and IV collagen-binding adhesin, laminin and dentin. Its equivalent in *E. faecium, acm* (70.0%), is responsible for the adhesin binding type I collagen and, to a lesser extent, type IV collagen [41,44,45]. Although the *acm* genes are common in *E. faecium*, they are not expressed in all bacteria. In many cases, mutations render genes inactive [44,45]. Unfortunately, confirmation of the phenotypic activity of virulence genes exceeded the goal of the present study. Collagen binding most often concerns multidrug-resistant strains of clinical origin [44,45]. Genes encoding the adhesion antigens *efaAfs* (*E. faecalis*) and *efaAfm* (*E. faecium*) were found in all isolates, which significantly exceeded the values in the study of game isolates, where only 14.3% of *E. faecalis* contained *efaAfs* and 8.9% of *E. faecium* had the *efaAfm* gene [37]. Factors encoded by other detected virulence genes are also important in the spread of infection, such as: ElrA protein, which prevents the detection and chemotaxis of macrophages towards bacteria [46,47]; and sex pheromones (*cCF10, coOB1, cad, camE*), signaling the start of conjugative material transfer between cells [43,48]. At the same time, secreted enzymes play a key role: hyaluronidase (*hylA* and *hylB* genes), damaging host tissues; gelatinase (*gelE* gene), hydrolyzing gelatin; casein, hemoglobin and other peptides; and cytolysin (*cyl* genes), with bactericidal and cytolytic activity [8,9]. Strains producing cytolysin are characterized by greater virulence and are more often found in samples of clinical origin [8,9,32,39]

Plasmids play a key role in the spread of resistance and virulence genes, not only within the genus but also beyond it [4,14,15]. Our finding of the repUS43 replicon location on the same contig as the *tet(M)* and *tet(L)* genes in all tetracycline-resistant isolates may suggest the possibility of horizontal spread of these resistance genes. In addition, in some strains, the genes encoding the production of an aggregating substance and the secretion of cytolysin were associated with the presence of the rep9a replicon. The presence of resistance and virulence genes located on the same contig as the replication genes of plasmids suggests that they can be carried by plasmids; however, in order to confirm this relationship, further research should be performed to assemble individual plasmids.

## 5. Conclusions

The high diversity of *Enterococcus* species and their sequence types of wild bird origin overlap with similar observations in other animals, the environment, and humans, confirming the adaptation of these bacteria to a broad range of hosts. The presence of strains containing numerous genes determining virulence factors as well as plasmids, and the presence of, albeit in a small percentage, isolates resistant to antimicrobial agents, provides an insight into free-living birds as potential reservoirs and vectors of *Enterococcus* and resistance determinants, with possible public and animal health impact.

## Figures and Tables

**Table 1:** Distribution of Minimal Inhibitory Concentrations of *Enterococcus faecium* (*n* = 10). White zones display applied antimicrobial dilution ranges (mg/L). Red vertical lines indicate interpretative criteria (EUCAST) epidemiological cutoff values (ECOFF). NWT (non-wild type) defines isolates carrying antibiotic resistance mechanisms; that is, MIC value higher than epidemiological cutoff value.

Antimicrobial Name	NWT	Minimal Inhibitory Concentration Value (mg/L)																
	%	0.032	0.064	0.12	0.25	0.5	1	2	4	8	16	32	64	128	256	512	1024	>1024
Ampicillin	0.0%					2	5	3										
Chloramphenicol	10.0%								2	7			1					
Ciprofloxacin	10.0%						2	1	4	2	1							
Daptomycin	0.0%							5	5									
Erythromycin	10.0%						1	5	3					1				
Gentamicin	10.0%									9								1
Linezolid	0.0%						1	9										
Quinupristin/dalfopristin	60.0%					1	3		5	1								
Teicoplanin	0.0%					10												
Tetracycline	10.0%						9							1				
Tigecycline	0.0%		6	2	2													
Vancomycin	0.0%						8	2										

**Table 2:** Distribution of Minimal Inhibitory Concentrations of *Enterococcus faecalis* (*n* = 50) White zones display applied antimicrobial dilution ranges (mg/L). Red vertical lines indicate interpretative criteria (EUCAST) epidemiological cutoff values (ECOFF). NWT (non-wild type) defines isolates carrying antibiotic resistance mechanisms; that is, MIC value higher than epidemiological cutoff value.

Antimicrobial Name	NWT	Minimal Ipnhibitory Concentration Value (mg/L)																
	%	0.032	0.064	0.12	0.25	0.5	1	2	4	8	16	32	64	128	256	512	1024	>1024
Ampicillin	0.0%					6	26	18										
Chloramphenicol	0.0%								4	46								
Ciprofloxacin	0.0%				1	4	40	5										
Daptomycin	0.0%					5	28	16	1									
Erythromycin	0.0%						25	22	3									
Gentamicin	0.0%									10	37	3						
Linezolid	0.0%						4	46										
Quinupristin/dalfopristin	0.0%								2	37	11							
Teicoplanin	0.0%					48	2											
Tetracycline	0.0%						44	1					3	2				
Tigecycline	4.0%		9	23	18													
Vancomycin	0.0%						29	18	3									

## Data Availability

The sequences were deposited at the GenBank under the BioProject number PRJNA939505 (*E. faecium*) and PRJNA940637 (*E. faecalis*).

## Supplementary Materials

The following supporting information can be downloaded at: https://www.mdpi.com/article/10.3390/pathogens12060836/s1, Table S1: New sequence types (ST) confirmed among *E. faecium* isolates. New variants of alleles were bolded, Table S2: New sequence types (STs) confirmed among *E. faecalis* isolates. New variants of alleles were bolded.
