# Supplementary material for: Prevalence of Enterococcus spp. and the Whole-Genome Characteristics of Enterococcus faecium and Enterococcus faecalis Strains Isolated from Free-Living Birds in Poland
Source: Pathogens. 2023 Jun 16;12(6):836. doi: 10.3390/pathogens12060836 (PMC10305306; doi:10.3390/pathogens12060836)
Supplement: Supplementary file 1 [file pathogens-12-00836-s001.zip › pathogens-2385790-supplementary.docx]

**Table S1.** New sequence types (ST) confirmed among *E. faecium* isolates. New variants of alleles were bolded.

| sequence ID | MLST | Alleles | | | | | | |
| --- | --- | --- | --- | --- | --- | --- | --- | --- |
|  |  | *atpA* | *ddl* | *gdh* | *purK* | *gyd* | *pstS* | *adk* |
| P_20_BEAA_132B | ST2342 | *atpA*(13) | *ddl*(16) | *gdh*(8) | *purK*(8) | *gyd*(6) | *pstS*(27) | *adk*(6) |
| 708B_2 | ST2341 | *atpA*(4) | *ddl*(5) | *gdh*(1) | ***purK*(167)** | *gyd*(1) | *pstS*(20) | *adk*(1) |
| 38B | ST2340 | *atpA*(13) | *ddl*(8) | *gdh*(8) | *purK*(8) | *gyd*(6) | *pstS*(51) | *adk*(11) |

**Table S2.** New sequence types (STs) confirmed among *E. faecalis* isolates. New variants of alleles were bolded.

| sequence ID | MLST | Alleles | | | | | | |
| --- | --- | --- | --- | --- | --- | --- | --- | --- |
|  |  | *gdh* | *gyd* | *pstS* | *gki* | *aroE* | *xpt* | *ygiL* |
| 683B_1 | ST1368 | *gdh*(15) | *gyd*(40) | ***pstS*(121)** | *gki*(54) | *aroE*(76) | *xpt*(15) | *yqiL*(11) |
| 750B | ST1370 | *gdh*(11) | *gyd*(6) | *pstS*(50) | *gki*(33) | *aroE* (1) | ***xpt*(111)** | *yqiL* (10) |
| 752B | ST1371 | *gdh*(27) | *gyd*(1) | *pstS* (40) | *gki*(32) | ***aroE*(126)** | *xpt* (26) | *yqiL* (31) |
| 755B | ST1370 | *gdh*(11) | *gyd*(6) | *pstS* (50) | *gki*(33) | *aroE* (1) | ***xpt*(111)** | *yqiL* (10) |
| 709B_4 | ST1369 | *gdh*(6) | *gyd*(2) | *pstS* (11) | *gki*(5) | *aroE* (10) | *xpt* (1) | ***yqiL*(118)** |
| P_20_BEAA_124B | ST1373 | ***gdh*(121)** | *gyd*(6) | *pstS* (11) | *gki*(45) | *aroE* (4) | *xpt* (20) | *yqiL* (28) |
| P_20_CHO_130B | ST1377 | *gdh*(9) | *gyd*(6) | *pstS* (4) | *gki*(49) | *aroE* (25) | ***xpt*(112)** | *yqiL* (61) |
| P_20_BEAA_136B | ST1374 | ***gdh*(122)** | *gyd*(7) | *pstS* (9) | *gki*(1) | *aroE* (1) | *xpt* (1) | *yqiL* (1) |
| P_20_BEAA_137B | ST1375 | *gdh*(3) | *gyd* (6) | ***pstS*(122)** | *gki*(12) | *aroE* (9) | *xpt* (10) | *yqiL* (7) |
| 20_CHO_157B_1 | ST1366 | *gdh*(56) | *gyd* (8) | *pstS* (31) | *gki*(8) | *aroE* (40) | *xpt* (16) | *yqiL* (13) |
| 20_BEAA_160B | ST1366 | *gdh*(56) | *gyd* (8) | *pstS* (31) | *gki*(8) | *aroE* (40) | *xpt* (16) | *yqiL* (13) |
| P_20_BEAA_217B | ST1376 | *gdh*(1) | *gyd* (7) | ***pstS*(123)** | *gki*(1) | *aroE* (1) | *xpt* (10) | *yqiL* (1) |
| P_20_CHO_226B_2 | ST1378 | *gdh*(81) | *gyd* (6) | *pstS* (7) | *gki*(5) | *aroE* (3) | *xpt* (2) | *yqiL* (20) |
